# Breathing through a troubled life – a phenomenological-hermeneutic study of chronic obstructive pulmonary disease patients’ lived experiences during the course of pulmonary rehabilitation

**DOI:** 10.1080/17482631.2019.1647401

**Published:** 2019-08-21

**Authors:** Charlotte Simonÿ, Ingrid Charlotte Andersen, Uffe Bodtger, Regner Birkelund

**Affiliations:** a Department of Physiotherapy and Occupational Therapy, Slagelse Hospital, Slagelse, Denmark; b Institute of Regional Health, University of Southern Denmark, Odense, Denmark; c Section of Nursing Science, Institute of Health, Aarhus University, Aarhus, Denmark; d Department of Internal Medicine M1, Slagelse Hospital, Slagelse, Denmark; e Department of Respiratory Medicine (M1), Naestved Hospital, Naestved, Denmark; f Department of Respiratory Medicine, Zealand University Hospital, Roskilde, Denmark; g Institute of Regional Health Science, University of Southern Denmark, Odense, Denmark; h Department of Health Research , Lillebaelt Hospital, Vejle, Denmark; i Institute of Regional Health Research, University of Southern Denmark, Odense, Denmark

**Keywords:** Rehabilitation, chronic obstructive pulmonary disease, patients’ experience, participant observation, interviews, phenomenological-hermeneutic design

## Abstract

**Purpose**: Exploring real-life experiences of Chronic Obstructive Pulmonary Disease (COPD) patients during rehabilitation can contribute with new knowledge of what has significance for their participation and chance for improved health and well-being. Therefore, this study aims to gain in-depth knowledge of COPD patients’ lived experiences while following standard pulmonary out-patient rehabilitation.

**Methods**: Combined participant observations and interviews were conducted among 21 participants in pulmonary rehabilitation. A three-leveled phenomenological-hermeneutic interpretation was applied.

**Results**: Living with COPD was challenging due to dyspnea and other physical troubles. This caused a lack of trust in the body and complicated rehabilitation participation. When improving management of breath during rehabilitation, the patients gained a new sense of trust in the body. This was accompanied by a nascent hope and increased well-being. However, not succeeding in this left patients with a persistent lack of hope.

**Conclusions**: Comprehensive troubles in living with COPD paradoxically prevents patients’ prospect of overcoming a perceived lack of trust in their body during standard pulmonary rehabilitation. Enhancing breath management has a significant impact on COPD patients’ trust in own capabilities to improve well-being and health. Future rehabilitation must accommodate COPD patients’ troubles by longer-lasting, well-coordinated, individually supportive and more easily accessible programmes.

## Introduction

This paper illuminates what is at stake for patients with chronic obstructive pulmonary disease (COPD) during the trajectory of a Danish standard out-patient rehabilitation.

COPD is a leading cause of disability and mortality and represents a considerable world-wide socio-economic burden. As estimated to be the third largest cause of death in 2030, there is an ongoing and global focus on improving treatment and management in pharmacological as well as non-pharmacological ways to COPD patients (Global Initiative for Chronic Obstructive Lung Disease, 2018; World Health Organization, 2018).

Pulmonary rehabilitation is an internationally acknowledged intervention, which is shown to be effective to improve dyspnea, health status, and exercise tolerance (McCarthy et al., 2015). Multidisciplinary pulmonary rehabilitation programmes are well-established and consist of several components that can be used to meet the goals and needs of the individual patient (Spruit, 2014). Along with exercise training, most programmes comprise educational components with the purpose of both increasing knowledge and improving skills required for effective self-management (Spruit, 2014). However, there are several barriers to both partial and full participation in pulmonary rehabilitation programmes (Cassidy, Turnbull, Gardani, & Kirkwood, 2014; Cox, Oliveira, Lahham, & Holland, 2017; Keating, Lee, & Holland, 2011; Rochester et al., 2015). Access issues and transportation challenges have been shown to be of major importance (Cassidy et al., 2014; Global Initiative for Chronic Obstructive Lung Disease, 2018; Keating et al., 2011; Rochester et al., 2015). Moreover, qualitative interview studies suggest that patients may decline pulmonary rehabilitation if they do not find the offer appropriate, if they find the offer as conflicting with daily activities, or if they perceive themselves too frail from COPD and comorbidities (Keating et al., 2011; Mathar, Fastholm, Lange & Larsen, 2017).

Previous qualitative interview studies have widely described that perceived breathlessness is the core challenge for COPD patients (Giacomini, DeJean, Simeonov, & Smith, 2012). Especially noteworthy, episodes with exacerbated breathlessness are reported to cause anxiety and stressed emotions, which can be hard to cope with (Bailey, 2004). Moreover, the situation for COPD patients can be complicated by frequent comorbidities. As such, the physical, -as well as the psychosocial burden-, can be significant to how COPD patients manage to take control of their illness (Cooney et al., 2013; Giacomini et al., 2012). Nevertheless, knowledge about how dyspnea, comorbidities, and additional inabilities affect patients’ participation in pulmonary rehabilitation is still sparse.

It seems, then, that the challenge is still to understand how to provide rehabilitation that is perceived accessible and meaningful for the patients. Yet, there is still a need for more knowledge of what is at stake to COPD patients while participating in pulmonary rehabilitation. A deeper knowledge of COPD patients’ lived experiences might clarify the nature of their challenges.

From the perspective of COPD patients, participation in pulmonary rehabilitation has mainly been described by interview studies. It is found that patients are supported to change their prior perceptions of dyspnea and panic, leading to increased confidence in physical and social activity in the immediate time just after (Williams, Bruton, Ellis-Hill, & McPherson, 2010). However, by incorporating new routines into daily living subsequently, the programme can present challenges (Meis et al., 2014). Likewise, the time after completion of the pulmonary rehabilitation is a phase characterized by uncertainty and vulnerability, which the patients associate with continual demands for self-management and decreased access to professional support (Halding & Heggdal, 2012). Hence, despite the documented benefits, it seems that COPD patients do not always perceive themselves prepared sufficiently to manage on their own. Therefore, it is warranted to explore what actually occurs in the sessions of rehabilitation and what is of significance to the patients during their rehabilitation process. It is anticipated to provide expanded knowledge about what is at stake to the patients by applying a study design that combines field observations and interviews (Simonÿ et al., 2018). By such a study detailed and nuanced knowledge of patients’ real-life experiences can be gained. This may contribute with important angles of perspective that can help health care professionals improve models for future rehabilitation programmes. This study, therefore, aims to gain knowledge of COPD patients’ lived experiences while participating in a standard out-patient rehabilitation programme.

## Materials and methods

The design of this qualitative study was guided by a phenomenological hermeneutic approach. A combination of ethnographic participant observations and narrative interviews was conducted to capture the patients’ lived experiences in-depth (Simonÿ et al., 2018). Data, in the form of gathered field notes (FN) and transcribed interviews (I), were interpreted with inspiration from the theory of narrative and interpretation by the French philosopher Paul Ricoeur (Simonÿ et al., 2018).

The present study is part of a larger research project that aims to develop new solutions within the field of rehabilitation to COPD patients with concern to what is essential to them.

### Setting and participants

The first author conducted the data generation in a Danish hospital, from August 2016 until March 2017. As shown in Table I, 11 men and 10 women participated in the study.

**Table I.** Characteristics of the participating patients.

							Exercise capacity (meters) with BORG-CR10 score			
Participant	Gender	Age (years)	Married/ cohabiting	Smoking	FEV_1_ (%)	mMRC score	Before	After	Attended Pulmonary Rehabilitation before	COPD-related hospitalization (admissions/ total days)
P1_(SPR)_	M	78	+	FS	25	3	120 (1/6)	-	Yes	1/4
P2_(SPR)_	M	84	+	FS	45	3	330 (0/3)	-	No	
P3_(SPR)_	F	76	+	FS	45	3	220 (0/4)	180 (5/5)	No	
P4_(SPR)_	M	71	-	FS	61	3	165 (0/6)	300 (-)	No	
P5_(SPR)_	F	64	-	FS	47	3	440 (1/3)	448 (3/4)	No	
P6_(SPR)_	F	75	+	FS	81	2	337 (0,5/3)	400 (2/5)	No	
P7_(SPR)_	F	65	-	FS	46	1	390 (0/4)	420 (0/-)	No	
P8_(SPR)_	M	71	-	S	39	3	180 (0,5/7)	150 (2/5)	No	
P9_(SPR)_	M	59	+	S	26	3	405 (0/5)	378 (6/5)	No	
P10_(SPR)_	F	80	-	FS	41	0	214 (0/5)	300 (0/9)	Yes	
P11_(SPR)_	M	64	+	FS	46	3	373 (0/4)	422 (5/-)	No	
P12_(SPR)_	M	82	+	FS	30	2	270 (0/4)	270 (-)	No	
P13_(SPR)_	M	82	+	FS	33	3	170 (0/6)	180 (-)	No	
P14_(SPR)_	M	65	-	S	32	2	236 (0/-)	420 (0/6)	Yes	2/6
P15_(SPR)_	M	71	+	FS	42	2	316 (0/3)	274 (-)	No	
P16_(SPR)_	F	71	-	FS	79	4	295 (1/3)	326 (0/3)	No	1/5
P17_(SPR)_	F	57	+	FS	43	0	480 (1/7)	360 (-)	No	
P18_(SPR)_	F	71	-	FS	65	2	215 (0/2)	327 (0/7)	No	
P19_(SPR)_	F	70	+	FS	34	4	215 (5/-)	262 (0,5/6)	No	
P20_(SPR)_	M	80	+	FS	48	3	160 (0/6)	180 (-)	Yes	
P21_(SPR)_	F	55	+	FS	66	2	510 (0,5/-)	487 (2/7)	No	
MEAN		71			46.4	2.4	283	320		

The patients from the standard pulmonary rehabilitation (SPR) were assigned codes such as patient 1 (P1) _(SPR)_ in alphabetical order.

M: male; F: female.

FEV_1_: Forced expiratory volume in 1 second.

mMRC: modified Medical Research Council dyspnea scale.

Smoking: S: Smoker, FS: Former smoker.

Exercise capacity: Was measured before and after the rehabilitation by 6 Minute Walk Test. Borg-CR10 was measured before and subsequently after the walking test.

COPD related hospitalizations were registered during the rehabilitation period. In total 4 patients were admitted to the hospital 4 times during rehabilitation due to COPD. In sum, those hospitalizations lasted for 15 days.

The patients’ overall attendance to the rehabilitation was 4 out of 5 times.

They were invited to participate if they were diagnosed with COPD, included in the pulmonary rehabilitation programme and spoke Danish. In total, 27 patients were suitable and invited to participate in the study. Of those 6 could not cope with the task of participating. The 21 included participants followed an eight-week long pulmonary rehabilitation programme in three similar but different groups. At random occasions, close relatives participated in the sessions. For further information about the programme, see Table II.

**Table II.** Outline of the standard pulmonary rehabilitation programme.

10–12 patients are enrolled in the COPD-teams, typically within 4 weeks after discharge from the hospital, if they have been admitted to the department of lung medicine or as part of an offer from the pulmonary outpatient clinic. A few days before the start they are invited to an introduction, at the hospital, where they perform start tests and have individual consultations with physiotherapist, nurse, dietician and occupational therapist. On this day they give consent to participating in the programme. The programme takes place in the hospital and consists of physiotherapist-guided exercises for one hour twice a week for eight weeks and two 3-hours sessions of psychosocial education content. The patients can bring their closest relatives to take part in these sessions.	
Content	Elements
Exercise-based sessions	Each exercise session consists of 10–15 minutes of warm-up activities followed by varied strength and endurance exercises. The exercises consist of e.g cycling, Nordic walking, ball and elastic exercises. The patients are instructed by the physiotherapist during the exercises, either group-based or individually. Each session ends with 5–10 minutes of warm-down with relaxation and stretching exercises. The patients often ask the physiotherapist questions about their illness during this part of the session.
Education sessions	1^st^ session was held during the first week: **12 pm—14 pm—**by a nurse Physiological and anatomical factors related to COPD and psychosocial reactions. **14 pm—15 pm:—**by an occupational therapist Strategies in order to prevent or minimize respiratory stress during activities. 2^nd^ session was held in week 3: **12 pm −13 pm**:—by a nurse Medicine and inhalation techniques **13 pm −14 pm**:—by a physiotherapist Exercise principles and respiratory techniques, e.g. positive expiratory pressure (PEP), Pursed Lip Breathing (PLB), coughing techniques and how to manage the symptoms and become physically active in daily life. **14 pm −15 pm**:—by a dietitian Nutrition and risk factors related to COPD. The patients are offered individual consultations with the healthcare professionals if deemed relevant.

The programme is planned according to the Danish National Guidelines for pulmonary Rehabilitation (Sundhedsstyrelsen, 2014). It is free of charge to the patients, and if needed, transport to the hospital is arranged without costs.

### Participant observations

In accordance with ethnographic methods (Hammersley & Atkinson, 2007; Spradley, 1980), participant observations were conducted during the entire rehabilitation period. The aim was to get insight into what happened in the sessions and how the patients responded to the programme. The researcher was continuously guided by the overall questions of *what was essential to the patients during their participation in the rehabilitation programme*. The long-lasting observations allowed a “bridled” approach with dwelling upon not only initially clear, but also more hidden phenomena, expressed by the actors (Dahlberg, 2006; Simonÿ et al., 2018). To achieve the most relevant data, the researcher was alternating between full participation and full observation (Hammersley & Atkinson, 2007; Spradley, 1980). Both informal and more structured explorational ethnographic interviews were conducted (Spradley, 1979). During the observations, notes and quotes were written in a notebook. Inspired by ethnographic research, field notes were made immediately after each of the 48 sessions and typed into a computer (Emerson, Fretz, & Shaw, 1995; Hammersley & Atkinson, 2007). Statements from occasionally present relatives were included to give nuances to the data. There was a constant reflexion on the data, which opened the awareness of what was seen, sensed, and heard, leading to what should be explored further (Hammersley & Atkinson, 2007).

### Interviews

Inspired by principles from phenomenological narrative interviews (Fog, 2004; Kvale, 1996), the participants were interviewed individually after their completion of the rehabilitation. A semi-structured interview guide with open questions was used. The aim was to gain more insight into the participants’ personal experiences by letting them express themselves with focus on what was of greatest importance to them. In an open, curious and trustful tone, they were encouraged to describe: *What living with COPD meant to them; How they experienced the rehabilitation programme; What it meant to them to participate in the programme* and *Which impact their participation had on their daily living*. They were invited to nuance their narrations during the interviews. In addition, follow-up questions were asked to further explore aspects of special interest from the participant observations. The interviews were performed as it was most suitable to each individual, in a quiet room at the hospital (*n* = 12), in their private homes (*n* = 6), or by telephone (*n* = 3). The interviews lasted for up to 60 minutes. The shortest interviews (three) lasted approximately 20 minutes. All interviews were audio recorded and transcribed into texts within a few days.

### Ethical considerations

The study has been approved by the Ethical Committee (SJ-559) and the Danish Data Supervisory Committee by Region Zealand (J. Nr. REG-071–2016). Their guidelines were followed. In addition, the ethical principles of the Declaration of Helsinki were followed (World Medical Association, 2013). Informed consent was given on behalf of oral and written information. It was emphasised that participation was anonymous and voluntary.

When patients in a serious life situation are observed over time and interviewed, it can be considered as a form of nakedness because they agree to dare being watched and heard in matters of their personal life (Løgstrup, 2010). Such a delicate area requires a trusting relationship between the researcher and the patients. Constantly this ethical claim was considered during data generation and analysis.

### Data analysis and interpretation

Field notes and transcribed interviews were gathered into one collective text of 313 pages and interpreted in a three-leveled process inspired by Ricoeur’s theory of narrative and interpretation (Dreyer & Pedersen, 2009; Pedersen, 1999; Ricoeur, 1976; Simonÿ et al., 2018). This approach is closely described by Simonÿ et al. (Simonÿ et al., 2018) and can be characterized as a dialectical phenomenological-hermeneutical interpretation that provides highly informative knowledge of real-life experiences of what it is like to be in the world as a COPD patient while participating in pulmonary rehabilitation. It includes an initial *naïve reading, structural analysis* and a *critical interpretation and discussion* of what the study contributes to our knowledge (Simonÿ et al., 2018).

**Figure 1.**
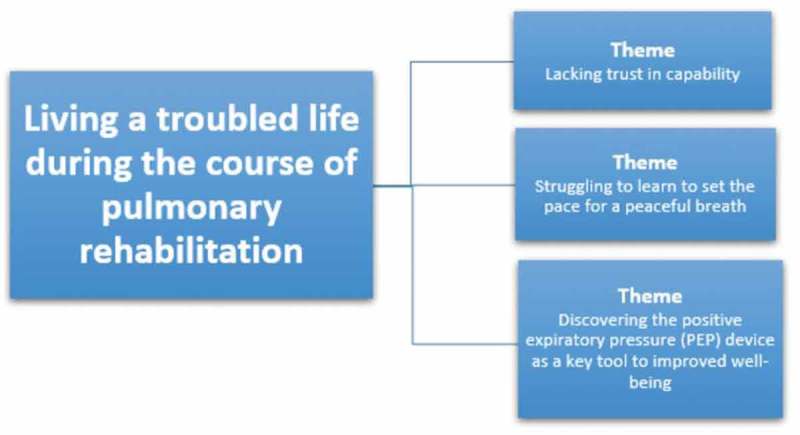
An illustration of the overall finding and the three themes. The description in the results reflects the themes. It is explained by the units of significance which draw upon all data from the units of meaning but is, however, presented by the inclusion of a few selected quotations from field notes, and interviews.

As illustrated in Table III the interpretation allowed for an appropriation of the world revealed in the data material and led to new knowledge of what is essential to the patients (Ricoeur, 1976). Dialectically moving back and forward between units of meaning from the text, identification of units of significance and themes was established. Figure 1 illustrates the themes.

**Table III.** A description of the three-leveled Ricoeur inspired interpretation of the coherent data material from both participant observation and interviews.

Based on the theory of interpretation by Ricoeur, (Ricoeur, 1976) and additional methodological reflections (Pedersen, 1999; Dreyer & Pedersen 2009; Simonÿ et al., 2018) the interpretation can be seen as a dialectical movement between the three levels in this table. The arrows indicate how there is a movement back and forth between the levels in the interpretation.

The naive reading is presented at the beginning of the following section of results, succeeded by the description of the identified themes from the structural analysis. These are explained by units of significance which is the authors’ interpretation of the data. To support the interpretation and ensure transparency, selected quotes from the field notes (FN) and interviews (I) are included. It is indicated by number Pn_(SPR)_ with reference to Table I, which participant is quoted.

The critical interpretation and discussion are described in the section of the discussion.

## Results

The overall impression from the naïve reading is that the patients’ lives were deeply troubled. The troubles were related to dyspnea but seemed also to be associated with several other limiting bodily inabilities. This appears to cause a sense of hopelessness. A hopelessness that appears to influence both the patients’ perception of life and their approach to the rehabilitation. Moreover, the troubles seemed to complicate the chances of fully participating in an engaging way in the rehabilitation. During the rehabilitation, some patients were guided in improving managing their breath, leading to invigorated well-being, confidence and hope. However, this was not gained for all.

### Lacking trust in capability

The dyspnea clearly sat the scene for the patients’ lives. It caused hopelessness and a restrained approach not only in everyday life but also in the rehabilitation.

Both observations and interviews covered that all the patients suffered from frequent episodes of dyspnea. This mediated panic and a fear of new upcoming attacks. One woman exemplified it in this way:

“*A few days ago, we went to a birthday party. I had a great time. I took on my coat and then it hit me, on my way to the car. I had an enormous attack, I could not breathe and I was shaking all over. I took my reliever and stayed in the car for more than half an hour, struggling to catch my breath. The rest of the evening was awful. I am not quite over it yet, I feel that I still have trouble breathing*.”(I,P3_(SPR)_).

The narration indicates, in line with other statements, that the episodes of dyspnea were experienced as difficult events that gave rise to long-lasting negative reverberation in body and mind. Thus, it was an intertwined part of their state-of-being that toned up a lack of trust in their own bodily capacity. The mere expectation of overloading themselves could cause dyspnea and a feeling of despair and powerlessness. Moreover, it obviously limited their persistence in the exercises during the rehabilitation, which was widely observed. “*It is like a reflex. You simply just stop yourself*”(I,P13_(SPR)_), a man said further explaining that he, like others, frequently held himself back during exercise to avoid the unpleasant dyspnea.

Often challenging dyspnea was prominent among the patients not only during exercise but also before the sessions started, as described in this field note:

“One of the men enters the waiting room with a loud sound of congested breath. He makes the pursued lips breathing and collapses on the sofa when reaching it. With a stuffy look in his eyes, he leans upon his knees and makes deep noisy dimpling breaths. He rises and leans upon the partition, struggling with catching his breath. He sits down again. His hands on his knees. After 8 minutes, the sound of his breath slowly decreases and by the 11th minute, he sits straight, taking off his jacket. He waits another five minutes before he enters the training hall.”(FN,P4_(SPR)_).

This typical episode illustrates how it was a struggle for the patients merely to attend the hospital. In addition, they explained that they often held themselves back in the exercises because they feared running out of energy capacity to return safely back home. Thus the dominance of dyspnea clearly prevented the patients from engaging fully in the rehabilitation.

The data also revealed that not only dyspnea was what troubled the patients. Apparently, many of them felt fatigued and fragile due to diverse infections. They considered themselves as being in danger of getting re-infections and had doubts about whether their body could endure the exercises. Thus, they quite often made cancellations of rehabilitation sessions. Furthermore, some missed sessions because they were admitted to a department of respiratory medicine with worsening lower respiratory symptoms (Table I). One woman explained: “*When I am sick I am totally out of energy. I am not able to exercise. I am only able to do one thing: ‘fight for staying alive.’”*(I,P16_(SPR)_). This quotation covers how episodes of illness could leave the patients tired and fully occupied with doubt whether they would survive. Hence, mental surplus to participate in rehabilitation was lacking. Nevertheless, some attended rehabilitation despite having pneumonia or lower urinary tract infection. This was clearly exhausting to the patients.

Along the course of the rehabilitation, it was observed that patients with musculoskeletal pain in neck, hips or ribs resigned from exercise. They disclosed that this was due to a fear of provoking additional pain.

Furthermore, other illnesses sometimes made participation in the rehabilitation impossible. In one exercise session, one female with diabetes experienced a hypoglycemic episode and thus had to leave the hospital and return home. At another session, a male was acutely admitted to the Department of Cardiology due to angina. Moreover, a man diagnosed with arterial fibrillation explained in an interview:

“*My coagulation system came out of control. Then there was a hitch somewhere. I was admitted to the department of cardiology. I missed three sessions of exercise on this account*.”(I,P12_(SPR)_).

These examples illustrate how other illnesses could turn everything upside down and pull patients away from the rehabilitation.

Even when attending rehabilitation sessions, the patients often missed important parts. It was observed that due to frequent tours to the lavatory or widescale coughing they often could not participate engagedly. Moreover, sleeping disorders made several attend, lethargic and dizzy to the sessions of exercise and education. On this account not only knowledge about e.g. medication, exercises, energy—saving techniques were missed but also invitations for individual consultations with e.g. the dietician or the nurse were lost.

Clearly, the dominance of dyspnea and other inabilities troubled the patients in ways that included a lack of capability and a sense of hopelessness. Accordingly, participating in rehabilitation was perceived more as a burdening hassle rather than a desired proposition.

### Struggling to learn to set the pace for a peaceful breath

Both observations and interviews reflected clearly that being able to cope with and diminish dyspnoea was crucial to the patients because it gave them a sense of being able to control both body and mind and thus achieve confidence.

Before starting on the rehabilitation course, one man stated: “*I have hopes that I can get over the edge and actually trust that I can catch my breath. That would be so nice*.”(FN,P8_(SPR)_). The quote underlines a desire for being able to improve the management of breath control. It also reflects that the patients considered it a difficult, yet crucial barrier that they could only overcome by a demanded effort.

Clearly, learning to manage breathing technique was a central issue during the rehabilitation. Thoroughly the physiotherapist encouraged the patients to work with breathing techniques. “*Inhale deeply through the nose, hold it a little and then exhale carefully through a small opened mouth*” they often said. (FN). These instructions were repeatedly given during exercising and were elaborated in the educational sessions. This inspired the patients to endeavor to try to learn to control their breath to a level of appropriate pace. They expressed a longing for learning this and made an effort to follow the instructions. This was not easy and it took a lot of practice and close guidance to “break the code”. Some patients succeeded in doing this, while others did not.

For those who succeeded, it was experienced to help them gain a sense of control and thus considerably improve a balance and trust in the body. Especially noteworthy, adequate performance of the breathing techniques helped the patients to eliminate both bodily discomfort and mental insecurity, as one woman disclosed:

“*When I am really bad, I use the technique that they taught me. I sit down saying to myself: now just calm down. I breathe through the nose. Then it comes to me: The peace*.”(I,P3_(SPR)_).

This description covers how managing the techniques were novel to the patients because it enabled them to remove panic and fear in favour of an appreciated sense of control and confidence.

Moreover, sufficient breath management obviously helped the patients in being able to exercise—often more than they had expected.

However, failing to manage these techniques clearly caused despondence and frustration. One man described this in the interview after completing the rehabilitation:

“*I am vexed because I am not able to control my breathing troubles when I am physically active. I have acquired a sustained limitation in my life. I feel like an old locomotive*.”(I,P1_(SPR)_).

Another stated: *“Unfortunately, my breath has not become better. Accordingly, my expectations have faded*.”(FN, P13_(SPR)_). These statements illustrate how not succeeding managing breathing techniques made the patients prone to feeling yet again dominated by the dyspnea and thus increasingly discouraged.

The data material revealed that, learning to manage the breathing techniques led to a feeling of regaining trust in the body. It improved the patients’ ability to manage exercising, set a pleasant pace for daily living and even to find inner peace in emotionally troubling situations. This caused more courage and hope for the future. In contrast, it was remarkable that patients who did not fully learn the technique maintained a lack of trust in the body and thus in their overall capability.

### Discovering the positive expiratory pressure (PEP) device as a key tool to improved well-being

During the sessions of rehabilitation, the observations reflected that the patients were quite unfamiliar with using the device for positive expiratory pressure aka the “PEP device” in daily speech. Over the span of the rehabilitation, the physiotherapist and nurses frequently told the patients to breathe in and out calmly and in a relaxed way through the 12 cm plastic T-tubed PEP device for10 breaths.

Moreover, special cough techniques were introduced and it was explained to the patients that this was to enable them to remove mucus from the lungs. They were instructed in repeating these sequences 3 times or more if needed. Furthermore, it was recommended to use the PEP device approximately 2–3 times a day as a part of their daily routines. Several patients stated that they had earlier been struggling to learn exactly how to manage this PEP device. Moreover, it was notable that some did not even know of the existence of the PEP device before attending the rehabilitation.

The patients valued the given guidance in hands-on exercising, as one woman said: *“I have asked for many instructions because I needed to know exactly how to manage the PEP device. That was how I learned to manage. And this is precious to me*.”(I,P5_(SPR)_). This quotation exemplifies that even though the patients acknowledged that it was a simple tool, they found it very complex to learn to use. It was thus conspicuous how they needed intensive instructions in this process.

Furthermore, it was remarkable how using the PEP device made it possible for the patients to improve their breath themselves when needed. A male patient returned after a weekend and described how by using the PEP device he had been able to loosen some mucus, coughing it up and thereby achieving highly improved breathing. With a big smile on his face, he said: *“This PEP device is quite a good invention.”* (FN, P11_(SPR)_). This situation reflects how the PEP devices became a beneficial key by which the patients were able to avoid stagnation of mucus and fill their lungs with fresh air, leading to what one patient named as “*being loosened up”*(I,P10_(SPR)_). This phrase emphasizes how the device could support unlocking not merely impeded breath, but also a renewed bodily confidence. In a compelling way, this device provided much more than a categorial effect: The patients learned to use it as an effective key to preparing themselves and being bodily able to perform in certain cases. Accordingly, using the PEP device made them more capable of activity.

Routine exercising with the PEP device made the patients realize that they could achieve a surplus of energy and thereby improve their daily life. Some stated that they felt that it caused an increased medical effect, leading to an overall better breath and bodily well-being.

However, some patients were continuously fighting to learn to use the PEP device without luck. The following field note reflects this:

“One of the men describes that he had been struggling to get rid of mucus by using the pep device at home. He looked at me with lifted eyebrows while shaking his head from side to side and said: *“I fail to do so, and it makes me tired of it all*.” (FN, P20).

This underlines that not fulfilling learning to manage the PEP device made the patients disheartened. To some extent, it gave rise to an increased feeling of hopelessness.

In sum, learning to exploit the PEP device obviously enabled the patients to loosen up not only their breath but also life itself. This way the PEP device became a significant key that enabled them to noticeable better management of their bodily troubles with a far-reaching positive impact on their lives. Not achieving this skill was on the other hand associated with renewable bodily hazzles and thus annoyance and intensified hopelessness.

## Discussion

This study provides new insight into how multifaceted troubles challenge COPD patients when following an eight-week long standard out-patient rehabilitation programme in Denmark and that support to gain a new trust in one’s body and capability by breath management is crucial, however difficult to achieve. In the following, this will be discussed with the inclusion of other research as well as in the light of the philosophy of bodily existence and education.

### Challenges to participate in the rehabilitation

An important finding in this study is that being troubled by not only dyspnea but also multiple other inabilities of the body seem to be of significance to the COPD patients’ participation in the standard rehabilitation programme. This study illustrates that the patients missed important aspects in the rehabilitation caused to dyspnea, fatigue and other symptoms of COPD along with other diseases and inabilities.

COPD patients’ suffering from dyspnea is well-documented in literature (Gysels & Higginson, 2008; McMillan Boyles, Hill Bailey, & Mossey, 2011; Williams, Bruton, Ellis-Hill, & McPherson, 2007). Cooney et al. have shown that breathlessness shapes the lives of COPD patients and governs their capacity. This causes a constant striving for balancing life in a co-existence with breathlessness (Cooney et al., 2013). Moreover, it is previously described how an exacerbation in COPD for a long time could make patients vulnerable and lead to increased levels of limitations and uncertainty (Giacomini et al., 2012). In this light, the suffering from impaired lungs is likely to lead to this being considered the overall dominating trouble of the patients’ lives. However, it is a significant contribution to clinical knowledge that dyspnea directly limits COPD patients’ participation in rehabilitation. Moreover, it is clear that multiple other inabilities add troubles to the patients during rehabilitation.

The findings in this study suggest that sleeping problems can make the patients feel unrested, dizzy and mentally affected. Likewise, another qualitative research shows that the experience of insomnia by patients with moderate to severe COPD is expressed to impose significant distress, which reduces their daytime functioning (Kauffman et al., 2014). Accordingly, fatigue appears to be a rather common challenge, and Stridsman and colleagues describe in their qualitative study that accompanying fatigue can be overwhelming in COPD (Stridsman, Lindberg, & Skär, 2014). Nevertheless, fatigue tends to be unexpressed to healthcare professionals and relatives (Stridsman et al., 2014). The present finding that fatigue reduces the patients’ benefits from rehabilitation underlines that this can be considered a significant problem, yet, underestimated in planning and carrying out the programmes.

It is documented in a prospective study that 62% of COPD diagnosed participants in pulmonary rehabilitation also suffered from comorbidities like diabetes, coronary heart disease and osteoporosis (Crisafulli et al., 2010). The present study reveals how patients experience such comorbidities to pose a considerable threat to their chances of participation in standard rehabilitation. Other studies have identified multiple barriers to rehabilitation attendance like: that transportation challenges (Keating et al., 2011), missing invitation, perceived overall frailty and a view of rehabilitation as a time-consuming offer that tends to conflict with daily activities (Mathar, Fastholm, Lange, & Larsen, 2017), attitudes, behavior, and beliefs about consequences (Cox et al., 2017). Since our findings show comprehensive troubles experienced by the patients during the trajectory of rehabilitation, the challenges of rehabilitation participation seem even more complex. Patients losing crucial information in educational sessions as well as the mere chance of engaged participation in exercising is a considerable challenge that calls for adjustments in rehabilitation planning. New studies of rehabilitation programmes that consider these multifaceted challenges of the COPD patients’ chances of participation can add important knowledge of how these troubles can be met. For example, more accessible solutions as e.g. telerehabilitation are likely to raise feasibility and thus outcome (Hoaas, Andreassen, Lien, Hjalmarsen, & Zanaboni, 2016; Holland et al., 2013; Zanaboni, Hoaas, Aarøen Lien, Hjalmarsen, & Wootton, 2016).

### A potential for gaining new trust in one’s capability

In our study, dyspnea and the multiple other physical inabilities implied a lack of trust in one’s body and capability. According to the French philosopher Merleau-Ponty, the body in every situation has already sensed and made sense before a conscious reflection or thinking can take place. He emphasizes that body and mind are inseparable and that ‘ the bodily cogito‘ is the basis of one’s condition of existence (Merleau-Ponty, 2002). In the line of this view, the hopelessness that is found to be dominant in relation to the malfunctioning body becomes a state-of-being, which can be considered an existential challenge to the patients.

By the present study it is highlighted that this state-of-being affects the patients’ approach to not only the rehabilitation but also to life itself. This existential dimension can bring new requirements to the multidisciplinary organization of pulmonary rehabilitation. Recognizing their life situation as troubled by lack of trust in one’s capability and missing hope can inspire to introduce more room for inviting them to share their inner feelings and concerns and accordingly support them in the individualized management of this. To do this, an intensive collaboration between nurses, occupational therapist and physiotherapist is needed together with targeted care. Furthermore, coordination between the hospital and municipal staff could improve such care for the patients in the overall course of the complicated and often long-lasting down-going vicious spirals that COPD patients are well-known to suffer from (Giacomini et al., 2012; Jackson, Oelke, Besner, & Harrison, 2012; Wodskou, Høst, Godtfredsen, & Frølich, 2014).

This study finds that the patients are closely supported in controlling their breath by using the PEP device and breathing technique. It should be noted that the patients struggle hard to learn to manage these breathing techniques and not everyone succeeds in this. Of novel interest, this becomes an important education for them with an enormous impact on their lives, leading to a renewed trust in the body together with improved confidence and hope. Thus this part of the rehabilitation gives rise to a more positive state-of-being for the COPD patients that should be of specific concern.

### Education towards invigorated hope, health and well-being

The Brasilian professor in education Paulo Freire, who introduced the concept of empowerment, points out that hopelessness can be replaced by hope through education. He states that education can give rise to:“. *nascent hope [that]coincides with an increasingly critical perception of the concrete condition of reality*” (Freire, 2005, p. 10) which frees human beings (Freire, 2005). With reference to this theory, it seems that the patients who learn to control and improve their breath are supported to act in order to help themselves in accordance with their specific reality. Especially because the dyspnea imprints the patients’ lives comprehensively, being able to improve their breath is clearly of high significance to their health and well-being. This study shows that improved breath management has a far-reaching positive impact on the patients’ lives, leading to an appreciated feeling of being loosened up.

Moreover, following Freire’s theory, it seems that the patients are given a nascent hope in the rehabilitation when they succeed in controlling their breath. A hope that supports them in developing critical perception and consciousness about their breathing difficulties, leading to the ability to help themselves to enhanced confidence in everyday life, feeling freer as a human being. This benefit is likely to affect the mere rehabilitation experience for COPD patients positively. It might also improve their chance of postponing the vicious downgoing spiral taking over in their lives and thus improving health.

In contrast, it also means that those who do not become able to manage breath control are missing such nascent hope, sense of freedom and rehabilitation benefit. Knowing that COPD patients’ courses across different health care sectors are unstructured (Giacomini et al., 2012; Wodskou et al., 2014), this further underlines that breath management must be strived at insistently for all patients, perhaps in a longer lasting and more well-coordinated approach to the health care support of these patients.

## Methodological considerations

Considering that important aspects of COPD patients’ lived experiences during their participation in rehabilitation might be blurred, the combination of participant observations and interviews is crucial to achieving sufficient in-depth knowledge. Moreover, the bridled approach used in the data generation ensured that the patients’ experiences were investigated in an open way that allowed the researcher to actively wait for the phenomena to show themselves. By this attitude, it became possible to untie firm intentional threads and achieve the elbow room that was needed to see, hear and sense the expressions made by the patients (Dahlberg, 2006). Hence, the data material included not only the patients’ verbal expressions but also observations of the context that they were part of, with bodily expressions and interactions. This permitted analysis and interpretation that reflected real-life with a diversity of nuances detailing how lived life was experienced (Simonÿ et al., 2018), adding new insight to the existing knowledge.

Moreover, this type of data opens for insight into a movement in the patients’ self-image. Focus on this particular part has been interpreted and described in another publication adding new knowledge about how a rewarding peer-fellowship increase COPD patients’ engagement in the rehabilitation leading to improved illness management (Simonÿ, Andersen, Bodtger, & Birkelund, 2019). However, more studies of new solutions within rehabilitation to COPD patients is warranted to better target their particular situation.

With regard to the applied analysis and interpretation, it is important to note that there is always more than one way to interpret a text (Ricoeur, 1976). To refine this work, all authors have participated and contributed with sustained critique during all the steps of the analysis. This enabled us to constantly confirm that the interpretation reflected reality in the best possible way. Other colleagues in the field have moreover discussed the findings critically.

## Conclusions

This study illustrates that COPD patients struggle with comprehensive troubles that prevent them from full and engaged participation in out-patient pulmonary rehabilitation, resulting in limited benefits from a standard eight-week rehabilitation programme. Patients with COPD are commonly impaired by symptoms related to other chronic illnesses, too, as COPD is frequently a part of multimorbidity. Thus, future rehabilitation programmes should address the overall impairment of multimorbidity and not only disease-specific symptoms.

However, improved breath management significantly enhances COPD patients’ trust in their own capability and well-being and should be a cornerstone in future rehabilitation.

In order to prevent the complex downgoing spirals of COPD, longer-lasting and more well-coordinated rehabilitation should be considered to this population of a more easily accessible and more individualized character.
